# A Retrospective Study of Chronic Post-Surgical Pain following Thoracic Surgery: Prevalence, Risk Factors, Incidence of Neuropathic Component, and Impact on Qualify of Life

**DOI:** 10.1371/journal.pone.0090014

**Published:** 2014-02-28

**Authors:** Zhiyou Peng, Huiling Li, Chong Zhang, Xiang Qian, Zhiying Feng, Shengmei Zhu

**Affiliations:** 1 Department of Anesthesiology, First Affiliated Hospital, School of Medicine, Zhejiang University, Hangzhou, Zhejiang Province, China; 2 Department of Thoracic Surgery, First Affiliated Hospital, School of Medicine, Zhejiang University, Hangzhou, Zhejiang Province, China; 3 Department of Anesthesiology, Perioperative and Pain Medicine, Stanford University School of Medicine, Stanford, California, United States of America; University of Washington, United States of America

## Abstract

**Background:**

Thoracic surgeries including thoracotomy and VATS are some of the highest risk procedures that often lead to CPSP, with or without a neuropathic component. This retrospective study aims to determine retrospectively the prevalence of CPSP following thoracic surgery, its predicting risk factors, the incidence of neuropathic component, and its impact on quality of life.

**Methods:**

Patients who underwent thoracic surgeries including thoracotomy and VATS between 01/2010 and 12/2011 at the First Affiliated Hospital, School of Medicine, Zhejiang University were first contacted and screened for CPSP following thoracic surgery via phone interview. Patients who developed CPSP were then mailed with a battery of questionnaires, including a questionnaire referenced to Maguire's research, a validated Chinese version of the ID pain questionnaire, and a SF-36 Health Survey. Logistic regression analyses were subsequently performed to identify risk factors for CPSP following thoracic surgery and its neuropathic component.

**Results:**

The point prevalence of CPSP following thoracic surgery was 24.9% (320/1284 patients), and the point prevalence of neuropathic component of CPSP was 32.5% (86/265 patients). CPSP following thoracic surgery did not improve significantly with time. Multiple predictive factors were identified for CPSP following thoracic surgery, including age<60 years old, female gender, prolonged duration of post-operative chest tube drainage (≥4 days), options of post-operative pain management, and pre-existing hypertension. Furthermore, patients who experienced CPSP following thoracic surgery were found to have significantly decreased physical function and worse quality of life, especially those with neuropathic component.

**Conclusions:**

Our study demonstrated that nearly 1 out of 4 patients underwent thoracic surgery might develop CPSP, and one third of them accompanied with a neuropathic component. Early prevention as well as aggressive treatment is important for patients with CPSP following thoracic surgery to achieve a high quality of life.

## Introduction

A surgical incision produces tissue injury, inflammation, and subsequent acute post-operative pain. Subsequent modulation of the peripheral and central nervous system can lead to chronic pain. Acute pain management including multimodal analgesia techniques aspires to stop the transition from acute pain to chronic pain, yet a significant reduction in the occurrence rate and severity of chronic pain has not occurred [1].

Thoracic surgeries including thoracotomy and video-assisted thoracoscopic surgery (VATS) are some of the highest risk procedures that often lead to chronic post-surgery pain (CPSP) [2]–[6]. Previous studies have shown that the prevalence of CPSP following thoracic surgery varies from 14% to 83% [2], [4], [6]–[11], while the prevalence of CPSP following thoracic surgery with neuropathic component varies from 22% to 66% [4], [6], [8], [11], [12]. Failure in proper management of CPSP following thoracic surgery is associated with higher morbidity and mortality, worse physical function and pain interference, and overall poor quality of life [13], [14]. Consequently, an increasing emphasis and effort has been focused on the mechanisms underlying the transition from acute pain to chronic pain, as well as aggressive prevention and management of CPSP following thoracic surgery.

In spite of the devastating nature of CPSP, the current understanding of CPSP following thoracic surgery and its potential neuropathic component remains poor. Based on the diagnosis criteria from the International Association for the Study of Pain (IASP) [10] and recent studies [9], CPSP following thoracic surgery is defined as “pain persisting for at least 3 months after surgery, pain is different from pre-operative pain, and pain caused by other conditions such as continuing malignancy or chronic infection should be excluded”. Some patients with CPSP following thoracic surgery also experience neuropathic component, which was recently redefined as “pain caused by a lesion or disease of the somatosensory system” [15]. According to the Assessment Committee of the Neuropathic Pain Special Interest Group (NeuPSIG) [16], diagnosis of neuropathic pain should be possible from history and clinical examination alone without the need for laboratory tests. Though a gold standard for neuropathic pain diagnosis is still lacking, some validated screening tools for neuropathic pain have become available, including a recently validated screening and diagnosis tool for neuropathic pain in Chinese patients [18]. In addition, a Chinese version of the Medical Outcomes Study Short Form-36 (SF-36) has also been validated and widely used to evaluate health-related quality of life (HRQOL) for patients who suffer from chronic post-operative pain or neuropathic pain [1].

This study aims to investigate the prevalence of CPSP following thoracic surgery and its neuropathic component, to identify risk factors, and to determine the effect of chronic pain on HRQOL following thoracic surgery. This information may be used to reduce the incidence of CPSP following thoracic surgery, guide the optimal post-operative pain management, and ultimately improve patients' qualify of life.

## Methods

### Ethics Statement

The study was approved by the ethics committee of the First Affiliated Hospital, School of Medicine, Zhejiang University. All subjects in this study were given verbal informed consent for their participation and the oral informed consent process was approved by the research ethical committee of the Department of Anesthesiology at the First Affiliated Hospital, School of Medicine, Zhejiang University. All participants received written information about the survey prior to the obtained consent. Written consent was deemed impractical in this large low risk survey and verbal consent was obtained by the interviewers and audited by the Department of Anesthesiology at the First Affiliated Hospital, School of Medicine, Zhejiang University.

### Subjects

A retrospective questionnaire survey was undertaken on patients who had undergone thoracotomy and VATS between 01/2010 and 12/2011 at the First Affiliated Hospital of School of Medicine, Zhejiang University, China. Criteria for exclusion include: age <18 years old, history of pre-operative chemotherapy, history of previous thoracic surgery, history of neurological disorders or thoracic trauma, emergent operation, and surgical site infection. The survey was conducted between 3/2012 and 6/2012, more than 3 months after the initial surgery. A total of 1624 patients were identified and contacted.

### Definitions and Questionnaire Design

According to the IASP [10] and recent studies [9], CPSP following thoracic surgery is defined as “persistent pain for at least 3 months after thoracic surgery, the pain differs in character from the pre-operative pain, and other causes for chronic pain such as continuing malignancy or chronic infection have been excluded.”.

The ID Pain questionnaire is a six-item, patient-completed screening tool designed to help differentiate nociceptive and neuropathic pain. Patients scoring greater than or equal to 2 on a Chinese version for ID pain questionnaire are considered to have neuropathic component [17].

The HRQOL was measured using a Chinese version of SF-36, which is a standardized and validated tool and has been widely used to evaluate patient's health. The SF-36 assesses eight scales: physical function (PF), role limitations due to physical problems (role physical, RP), body pain (BP), general health (GH), vitality (VT), social function (SF), role limitations due to emotional problems (role emotional, RE), and mental health (MH). The raw scores of each subscale were converted to a range from 0 to 100, with higher scores indicating better levels of functioning or well-being.

### Telephone interview and flow diagram

A total of 1624 patients were contacted. Patients with CPSP following thoracic surgery were identified if they answered yes to both of the following questions: 1) “Have you experienced any pain along the scar after surgery, different from what you had before the surgery?” 2) “Has the pain persisted for at least 3 months?”. Patients were then asked for permission to mail them a battery of questionnaires, including a questionnaire referenced to Maguire's research [1], [12], a validated Chinese version of the ID pain questionnaire [17], and a SF-36 Health Survey (Chinese version). Those patients who failed to respond within 1 month were reminded by telephone, and if necessary, were mailed the questionnaire again. Two months after sending the first questionnaire, the investigation was closed.

To identify potential risk factors for CPSP following thoracic surgery, patient demographics, past medical history, and clinical characteristics including anesthesia, surgery, and peri-operative pain management were collected from the electronic medical records system. Similar to a previous investigation [18], the following data were recorded: age, gender, presence of diabetes mellitus or hypertension, history of smoking, diagnosis of underlying thoracic disease, date of surgery, duration of the operation, surgical approach (VATS versus thoracotomy), surgeon, post-operative pain management (PCA regimen versus non-PCA regimen), the number of draining chest tube, duration of chest tube placement, surgical complications, cancer recurrence, and post-operative radiation and chemotherapy. Time since surgery was defined as the period between operation date and the telephone interview date. The operation time was defined from the moment of the incision to suture completion.

### Surgery, anesthesia, and post-operative analgesia

Four different surgical teams were involved in this study, with both thoracotomy and VATS. The thoracotomy approach used a conventional protocol without rib resection during the operation. Skin incision was parallel to the ribs at the lateral or posterior-lateral part of the intercostal space. After placement of an intercostal chest tube (JINGLE®, China, 30-Fr, straight thoracic catheter), the intercostal space was closed by pericostal sutures with extreme precautions applied to avoid the injury of intercostal nerves. The VATS approach used a uniform anterior three-port technique with standardized port placements regardless of the lobe to be resected or not. The camera port was made in the 7th inter-costal space at the middle axillary line, while the main port was placed in the 4th inter-costal space at the anterior axillary line, and the third port was placed in the 9th inter-costal space at the posterior axillary line. At the end of surgery, a draining chest tube (JINGLE®, China, 30-Fr, straight thoracic catheter) was inserted in the Camera port. The draining chest tube was removed as soon as no air leakage was detected for more than 24 hours, total amount of pleural effusion was less than 200 ml per day, and no chylothorax was present.

A total intravenous anesthesia (TIVA) technique composed of propofol, fentanyl or remifentanil, and intermittent rocuronium was used to maintain adequate anesthesia after induction. No paravertebral or epidural block was administrated preoperatively.

Under the current State Health Insurance Policy in Mainland China, cost related to PCA pump is not reimbursable. Many patients opted to choose the non-PCA regimen due to their willingness and capability for self-pay. As a result, 833 patients in our study chose PCA regimen for post-operative pain management. The PCA regimen included a patient controlled infusion pump (the Hospira GemStar® Pump, Hospira Inc., USA) programmed to deliver sufentanil with a continuous dose of 1–2 micrograms per hour, along with patient controlled bolus of 2–3 micrograms at a lockout interval of 10 minutes. The bolus dosage was subsequently adjusted and titrated for optimal analgesia (visual analogue scale (VAS) score less than 3). In addition, patients received intravenous Flurbiprofen at 50 mg every 12 hours for total 3 days, unless contradicted due to bleeding concerns or renal insufficiency. The non-PCA regimen included intravenous Flurbiprofen at 50 mg every 12 hours and tramadol 100 mg intramuscularly every 6 hours as needed (when VAS score more than 7). Those non-PCA option patients were managed by surgical teams from the Department of Thoracic Surgery, in contrast to the patients with PCA option who were managed by a dedicated Acute Pain Service (APS) team from the Department of Anesthesiology.

### Statistical analysis

Data was analyzed using SPSS 16.0 software (SPSS, Inc., Chicago, IL, USA). Continuous variables were presented as mean ± standard deviations and categorical data were shown as numbers and percentages. Comparison of continuous data was performed by Student's *t* test with normal distribution and by the Mann-Whitney U test for variables with non-normal distribution. The Chi-square test was used to compare groups with categorical variables. P value less than 0.05 was considered significant. Univariate analyses and multiple forward stepwise logistic regression analyses were performed to identify risk factors predicting CPSP following thoracic surgery and its neuropathic component. After univariate analyses, variables with *p* value less than 0.05 were included in a multivariate logistic regression analysis to identify the independent factors of CPSP following thoracic surgery and its neuropathic component.

## Results

### Characteristics of responders

**Figure 1:**
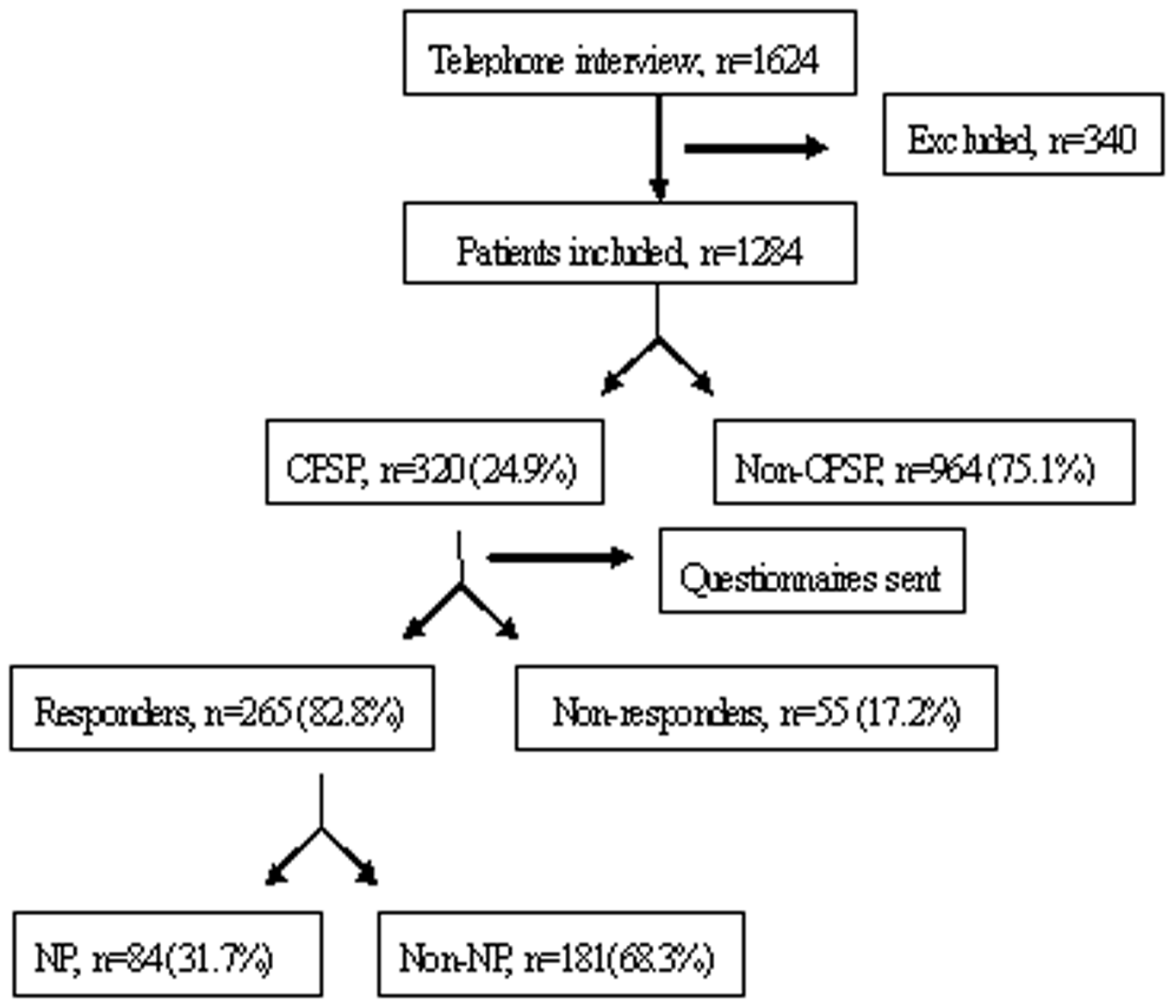
Flow diagram. CPSP, chronic post-surgical pain; NP, neuropathic pain.

As shown in the overall flow diagram of Fig. 1, 1624 total patients were contacted and evaluated for enrollment. After phone interviews, 340 patients were excluded because they were either deceased (n = 84), not answering phone calls (n = 186), refusing to participate (n = 19), or being diagnosed with a tumor recurrence at the time of interview (n = 51). We were able to collect and analyze the data from the rest 1284 patients, whose baseline characteristics were summarized in Table 1.

**Table 1:** Principle Characteristics of Patients with or without CPSP following thoracic surgery.

Participants	CPSP(*n* = 320)	Non-CPSP (*n* = 964)	*p* value
Age (<60 ys)	225 (70.3%)	619 (64.2%)	0.046
Female	147(45.9%)	305(31.6%)	<0.001
ASA physical status (I/II/III)	92/213/15 (29.0/66.6/4.7%)	277/630/54 (28.7/65.4/5.6%)	0.897
Smoking history	110(34.4%)	382(39.6%)	0.094
Hypertension	80(25%)	154(16.0%)	<0.001
Diabetes	14(4.4%)	26(27.0%)	0.134
Type of postoperative analgesia (PCIA/non-PCIA)	192/128 (60/40%)	641/323 (66.5/33.5%)	0.036
Type of pathology (cancer)	167(52.2%)	495(51.3%)	0.795
Type of procedure (Lung/Esophagus/Mediastinal)	256/28/36 (80/8.8/11.3%)	781/111/72 (81/11.5/7.5%)	0.055
Type of procedure (Thoracotomy/VATS)	239/81 (74.7/25.3%)	724/239 (75.1/24.8%)	0.859
Duration of operation (min)	143.02±70.76	141.11±75.26	0.690
Duration of chest tube drainage (≥4 days)	249(77.8%)	682(70.7%)	0.014
Number of pleural drains One	279(87.2%)	836(86.7%)	0.831
Two	41(12.8%)	128(13.3%)	
Follow-up (months)	11.75±5.373	11.57±5.834	0.627

Results are expressed as (n) or mean ± SD. CPSP, chronic post-surgical pain; NP, neuropathic pain, ASA indicates American Society of Anesthesiologists; PCIA, patient-controlled intravenous analgesia.

### Prevalence and properties of CPSP following thoracic surgery and its neuropathic component

**Figure 2:**
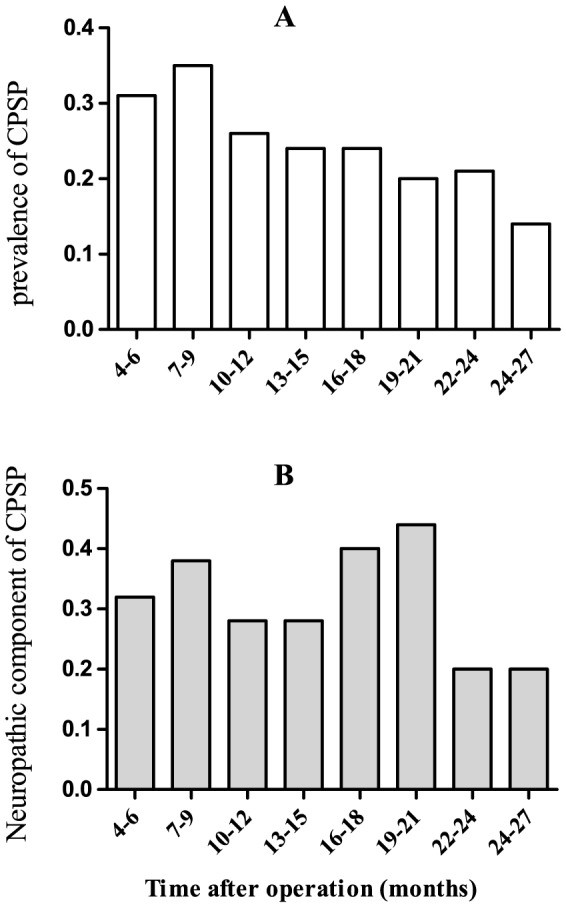
The trend for the prevalence of CPSP following thoracic surgery and neuropathic component of CPSP as a function of the length of time after operation. A: the trend for the prevalence of CPSP following thoracic surgery as a function of the length of time after operation, expressed in 3-month intervals. B: the trend for prevalence of neuropathic component of CPSP as a function of the length of time after operation, expressed in 3-month intervals. CPSP, chronic post-surgical pain; NP, neuropathic pain.

At the time of phone interview, the point prevalence of CPSP following thoracic surgery was 24.9% (320/1284 patients). Those 320 patients were then mailed with the questionnaire referred to Maguire's research to further explore the nature of chronic pain. 265 patients subsequently sent back the form, of which the point prevalence of neuropathic component of CPSP following thoracic surgery was found to be at 32.5% (86/265 patients). Figure 2 displayed the trend for the prevalence of CPSP following thoracic surgery and its neuropathic component as a function of the 3-month length of time after operation. As time elapsed after operation, it has a trend to fall for the prevalence of CPSP following thoracic surgery and its neuropathic component.

To assess the properties of chronic pain, the questionnaires referred to Maguire's research were sent to 320 patients with CPSP following thoracic surgery, and 265 replies were sent back. Among them, 73.5% of patients reported their pain being mild, 22.3% moderate, and only 4.2% severe. The most frequent descriptions of the pain include dull, throbbing, pressure, rebound tenderness, and needle-like and electric shock-like sensations around the surgical site and the chest tube placement sites. In addition, 67.3% patients did not feel improvement in their pain over time, while 25.7% reported that the pain was worse, especially when there is a rapid weather change, including cloudy or rainy days.

Significant differences were noted in terms of pain characteristics between CPSP patients with and without neuropathic component (Table 2). CPSP following thoracic surgery with neuropathic component have higher prevalence of moderate and severe pain than those without neuropathic component (*p*<0.05), as well as statistically significant increases in the incidence of unpleasant skin sensation (*p*<0.05), hyperalgesia (*p* = 0.013), and skin temperature change (*p* = 0.021)

**Table 2:** Responses to the questionnaire refer to the Maguire's research partially.

Question NO.	Question (in brief)	Answered ‘yes’ Total (n,%)	Answered ‘yes’ of Non-NP patients (n,%)	Answered ‘yes’ NP patients (n,%)	P value
2	Strange, unpleasant skin sensations	157(59.2%)	89 (49.7%)	68(79.1%)	0.000
3	Skin looks different	59 (22.3%)	42(23.5%)	17(19.8%)	0.498
4	Abnormally sensitive skin	137(51.7%)	83(46.4%)	54(62.8%)	0.012
5	Pain in sudden bursts	87(32.8%)	49(27.4%)	38(44.2%)	0.006
6	Skin temperature has changed	51(19.2%)	28(15.6%)	23(26.7%)	0.032
12	Improved with time	178(67.2%)	119(66.5%)	59(68.6%)	0.730
13	Pain severity mild	194(65.1%)	146(81.6%)	48(55.8%)	0.000
	moderate	59(29.1%)	28(15.6%)	31(36.0%)	
	severe	11(5.7%)	5(2.8%)	6(7.0%)	
14	Taking analgesia	56(17.7%)	43(24.0%)	13(15.1%)	0.096
15	Attended pain clinic	110(43.0%)	71(39.7%)	39(45.3%)	0.379
16	Pain the worst problem	104 (45.3%)	60(33.5%)	44(51.2%)	0.010
17	Pain limits daily activities	61(28.3%)	32(17.9%)	29(33.7%)	0.008
18	Pain limits sleep	72(32.5%)	39(21.8%)	33(38.4%)	0.008

NP, neuropathic pain.

### Risk factors for developing CPSP following thoracic surgery

Table 1 contains a list of the risk factors determined to be statistically significant for developing chronic post-thoracic pain, including age<60 years old, female gender, history of hypertension, lack of PCA regimen for post-operative analgesia, and prolonged duration of chest tube drainage (≥4 days). The prevalence of CPSP following thoracic surgery was significantly higher in patients below 60 years (225 patients, 70.3% versus 609 patients, 63.2%, *p*<0.05). In addition, women (147 patients, 32.5%) experienced more CPSP following thoracic surgery than men (173 patients 20.8%, *p*<0.01). CPSP prevalence in patients who had medical history of hypertension was also significantly higher than those without hypertension (*p*<0.01). The prevalence of CPSP in patients who were offered post-operative PCA based pain management was significantly less than those patients who did not receive PCA (*p* = 0.035). Finally, duration of chest tube drainage (≥4 days) was found to be a significant risk factor for CPSP after thoracic surgery (*p*<0.01).

No statistically significant difference was found between the CPSP group and non-CPSP group including ASA physical status, history of smoking, past medical history of diabetes, diagnosis of cancer, type of procedure (thoracotomy versus VATS), and the duration of the procedure (Table 1). In addition, no significant difference of CPSP prevalence was found among the four surgical teams (data not shown).

Based on logistic regression analysis, risk factors for developing chronic pain after thoracic surgery include age below 60 years old (OR: 1.51, 95% CI: 1.13–2.02), female gender (OR: 1.77, 95% CI: 1.36–2.31), hypertension (OR: 1.86, 95% CI: 1.35–2.57), lack of PCA for post-operation analgesia (OR: 1.31, 95% CI: 1.00–1.71), and prolonged duration of chest tube drainage (≥4 days) (OR: 1.55, 95% CI: 1.14–2.10).

### Predictive factors for the prevalence of neuropathic pain after thoracic surgery

Logistic regression was also used to determine the risk factor for neuropathic pain occurrence in patients who developed CPSP following thoracic surgery, including age, gender, ASA physical status, type of surgery, medical history of hypertension or diabetes, type of cancer, adjunct radiation and chemotherapy, number of chest tube drains, and duration of chest tube drainage. Interestingly, only the duration of chest tube drainage (≥4 days) was identified to correlate (positively) with neuropathic pain (*p*<0.05, OR: 0.421, 95% CI: 0.187–0.952).

### The impact of CPSP following thoracic surgery and neuropathic pain on daily living and quality of life

As shown in Table 2, the presence of a neuropathic component in patients with CPSP following thoracic surgery was associated with more severe pain. This subset of patients rate pain as one of their worst medical problems, has greatly limited their quality of life and has led to various sleep disorders (*p*<0.05). Interestingly, there was no significant difference in the proportion of patients with or without neuropathic component, in terms of visit to pain clinic and seeking additional pain medications (*p*>0.05).

**Figure 3:**
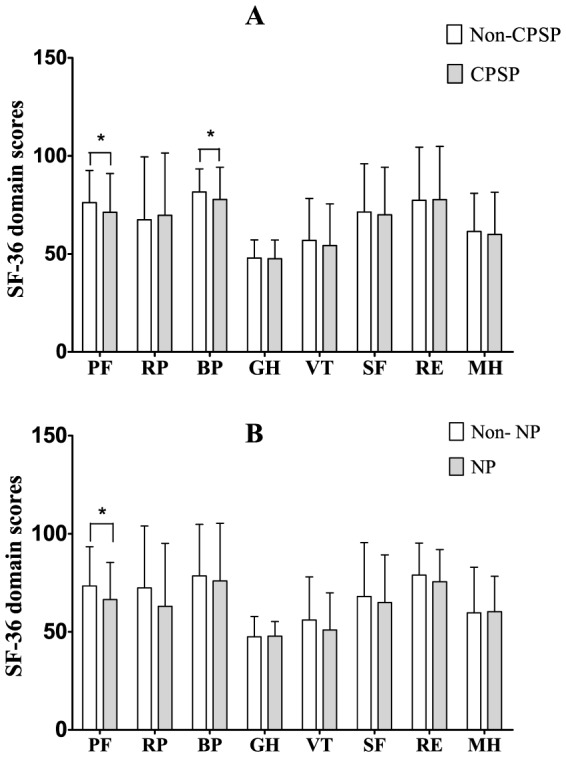
SF-36 domain scores of patients after thoracic surgery. Values of SF-36 domain scores represent mean ± SD in the respective group, whereas A show the result of SF-36 domain scores for the patients with CPSP or not, the B show the result of SF-36 domain scores for the patients with neuropathic pain or not. CPSP, chronic post-surgical pain; NP, neuropathic pain; PF, physical function; RP, role limitations due to physical problems; BP, body pain; GH, general health; VT, vitality; SF, social function; RE, role limitations due to emotional problems; MH, mental health.

In order to confirm whether CPSP following thoracic surgery and its neuropathic component negatively impacts quality of life, SF-36 Health Survey (Chinese version) questionnaires were mailed to both CPSP patients and non-CPSP patients (n = 600). Total 467 replies were received, among which 202 were non-CPSP patients, 179 were CPSP patients without neuropathic component, and 86 patients were CPSP patients with neuropathic component, respectively (Figure 3). Compared to non-CPSP patients, there was a significant decrease in PF score and BP score for CPSP patients (*p*<0.05). In addition, compared to CPSP patients without neuropathic component, there was a significant decrease in PF for CPSP patients with neuropathic component (*p*<0.05).

## Discussion

Our study found that one out of four patients undergoing thoracic surgery would develop characteristics consistent with persistent post-thoracic surgery chronic pain (CPSP), with one third of them reporting signs and symptoms consistent with neuropathic pain. Furthermore, our study has identified multiple risk factors for the development of CPSP following thoracic surgery, including age<60 years old, female gender, preoperative hypertension, post-operative non-PCA pain management regimen, and prolonged duration of post-operative chest tube drainage (≥4 days). In addition, the duration of post-operative chest tube drainage (≥4 days) was the only variable found to play a role for development of CPSP with neuropathic component. In our study, patients with CPSP have much worse quality of life, especially those with neuropathic component, suggesting the importance of preventing CPSP from development following thoracic surgery, as well as the need for aggressive treatment once it has developed. To our knowledge, this is the first study to investigate the prevalence of CPSP and its neuropathic component after thoracic surgery using a combined validated Chinese version of the ID pain questionnaire and a Short Form-36 (SF-36) Health Survey.

The reported prevalence of CPSP following thoracic surgery varies from 14% to 83% [2], [4], [6], [7], [10], [19] and the prevalence of its neuropathic component varies from 22% to 66% [4], [6], [8], [11], [12]. This wide range may be explained by discrepancies among studies in methodology, timing of assessment, surgical procedure details, and varying definitions used to assess CPSP following thoracic surgery or neuropathic pain [3], [4], [9], [20]. Unfortunately, it still lacks a standardized and universally accepted definition of CPSP following thoracic surgery [4], [9], [21]. The incidence of CPSP from our study appears lower in comparison with those reported in the literature [2], [4], [6]–[8], [11], [22], [23]. Possible reasons include different sample size (1284 total patients in our study, largest among all studies in the literature), different definitions of CPSP following thoracic surgery (≥3 months duration in our study compared to 2 months duration in studies from Deumens and Macare et al [9], [21]), different anesthesia techniques used for surgery [24] (TIVA in our study versus inhalational anesthesia in others) and different postoperative pain management [20], [25] (PCA versus non-PCA regimen).

In contrast, the prevalence of CPSP following thoracic surgery with a neuropathic component identified from our study (32.5%) is similar to findings in literatures [4], [8], [11]. Neuropathic pain, defined as “pain arising as a direct consequence of a lesion or disease affecting the somatosensory system” [15] can be a debilitating symptom. Several patient-reported measures of neuropathic pain have been developed, including the ID Pain questionnaire, Neuropathic Pain Scale (NPS), the Neuropathic Pain Diagnostic Questionnaire (DN4), the Leeds Assessment of Neuropathic Symptoms and Signs (LANSS), the Neuropathic Pain Questionnaire (NPQ) and others. Among these questionnaires, the ID pain questionnaire appears to accurately detect the presence of a neuropathic pain [17], [26], [27]

Although most of the patients who undergo thoracic surgery will experience some tissue and nerve injuries, not all will develop CPSP following thoracic surgery. A number of studies have attempted to identify potential risk factors for the development of CPSP and its neuropathic component, but come to variable results [10], [16], [27]. The pathogenesis of CPSP following thoracic surgery remains poorly defined, partially due to its multifactorial etiology. Consistent with previous studies [2], [3], [6], [8], [22], [23], [28], some of the reported preoperative ‘vulnerability factors’ have been confirmed in our study, including young age and female gender, which could have contributed to CPSP following thoracic surgery due to an exaggerated stress, inflammatory, and immune system response [9]. Young age is considered to be related to higher requirements for post-operative analgesia, stronger inflammatory and immune reaction, and increased neuroplastic responses [9], [23]. Gender related influences on pain and pain management have also been extensively studied in both basic and clinical researches [18], [29]–[32]. Compared to men, women not only have greater pain sensitivity to most experimental pain modalities displaying a higher level of acute postoperative pain, but also seem to be associated with a higher prevalence of chronic postoperative pain [18], [29]–[32]. In addition, no study has shown an increased risk of chronic postoperative pain in male patients [9]. Women are at particular risk for sensitization of their nervous system, as their neural circuitry tends to more readily detect pain and to attenuate it less than men [33].

Our study also for the first time demonstrated that pre-operative hypertension is a risk factor for CPSP following thoracic surgery. It is well known that there are adverse physiological effects of acute pain on cardiovascular systems by increasing sympathetic activity and stress response, which could be further exaggerated in hypertensive patients.

The trajectory for CPSP following thoracic surgery is not clear. Consistent to Steegers et al [8], we have found that the prevalence of CPSP following thoracic surgery did not decrease over time. The exact mechanism underlying the transition from acute to chronic pain is not clear, with possible mechanisms including central never system (CNS) reorganization and sensitization [9]. The etiology of CPSP following thoracic surgery is multifactorial with well-accepted predictors including intensity of acute post-operative pain [1], [2], [5], [9], [26], [27] and concurrent perioperative nerve injury [6], [18], [34]. A great deal of investigations found that the severity of acute postoperative pain was related to CPSP following thoracic surgery [1], [2], [5], [7], [9], [35]–[37], while others found no association [19], [23], [38], [39]. The underlying cause of this difference may lie in study design, using analgesic consumption instead of the acute pain scores [40], the different duration for acute pain evaluation [18], or a significant recall bias in retrospective investigations [18]. Our study found that post-operative non-PCA pain management regimen is a risk factor for CPSP. Possible reasons include improved post-operative pain relief when PCA regimen was used with systemic opioid [41]. The patients with the non-PCA regime usually were administered with tramadol intramuscularly at least every 6 hours as VAS score more than 7 except intravenous flurbiprofen 50 mg every 12 hours in our hospital. Multimodal pain management therapy with NSAIDs, COXIBs, or acetaminophen, regional blockade and/or opioid should be used and suggested strongly by the consultants and the American Society of Anesthesiologists because of its complicated mechanism and development of a chronic pain state after surgery [28], [41]. In these survey, the patients was not used with regional blockade or ketamine or pregabalin for acute pain management, and it was not well demonstrated that they can reduce the intensity of acute pain or the incidence of CPSP consistently [18], [23], [42]. The further investigation of multimodal post-operative pain management combined with NSAIDs, regional blockade and opioids should be taken in the future.

Intercostal nerve damage is well known as a main cause of CPSP following thoracic surgery [18], [43]. Both surgical incision and post-operation chest drain insertion are associated with nerve injury [44]. Consistent to previous studies [1], [2], [5], [6], [26], our investigation showed that the duration of chest tube drainage (≥4 days) (but not the different surgical procedures thoracotomy versus VATS, or different surgical site) was one of risk factors for CPSP following thoracic surgery. Chest drains are especially associated with movement-related pain which play a significant role in and lengthen the trajectory of acute post-operative pain until their removal [45]. Prolonged chest tube placement or inadequate post-operative pain management (the patient with non-PCA regimen) can be associated with more persistent pain, leading to continued stimulation to CNS and subsequent hypersensitivity and CNS reorganization. Moreover, Miyazaki demonstrated that chest tube insertion is also clearly harmful to both myelinated fibers (Aδ and Aβ fibers) and unmyelinated C fibers of the intercostal nerve and could result in post-thoracotomy pain by using current perception threshold testing recently [44]. This may explain why prolonged chest tube drains (≥4 days) were found to be not only a predictor for CPSP, but also the only predictor we identified for developing a neuropathic component of CPSP following thoracic surgery, which has not been reported before [4], [8], [11]. We should thus recognize that the draining chest tube would be removed as soon as possible, and more flexible drains should be applied and expected to eliminate or decrease the intercostal nerve injury and CPSP post-operatively [46].

Based on the SF-36 scores combined with scores from questionnaire referenced to Maguire's research, our study has found that CPSP patients in general have mild symptoms, with similar overall health vitality and mental health compared to patients who did not develop CPSP, though CPSP patients do have increased pain intensity and decreased physical functioning. Few CPSP patients needed to take pain medications. In contrast, the presence of neuropathic symptoms was associated with more severe pain, decreased daily activity, poorer sleep, and overall worse quality of life, which are consistent to findings from Steegers et al [8].

The retrospective study design is a limitation, and the shortcomings of self-reported pain on phone interview and in questionnaire surveys need to be kept in mind. We also could not rule out tumor recurrence and metastasis at the time point of phone interview and paper survey, which could complicate pain presentation. In addition, this is a single-center study; additional data from multi-center studies will be needed to verify our findings. Other potential risk factors including preoperative psychosocial comorbidity and genetic predisposition were omitted in this study as they could not be assessed or measured in retrospective study. Future studies are needed to address these limitations with well designed protocol [18], [20].

Chronic pain is a common complication from thoracic surgery, which can significantly impact patient's daily life. Our study showed that about one out of four patients who received thoracic surgery will develop persistent post-thoracic surgery chronic pain, with about one third of them has neuropathic component. There are several predicting risk factors identified from our study, of which the duration of chest tube drainage (≥4 days) seems to be essential for both CPSP following thoracic surgery with and without neuropathic component. Patients with the neuropathic component have significantly worse quality of life, suggesting the importance of preventing CPSP following thoracic surgery from developing as well as aggressive treatment of CPSP once it has developed. Further studies are needed to investigate the impact of minimizing risk factors on the incidence of CPSP, especially a multifaceted approach to shorten the chest tube drainage.

## References

[1] Kinney MA, Hooten WM, Cassivi SD, Allen MS, Passe MA, et al (2012) Chronic postthoracotomy pain and health-related quality of life. Ann Thorac Surg 93: 1242–1247. 22397986 10.1016/j.athoracsur.2012.01.031 PMC3442599

[2] Buchheit T, Pyati S (2012) Prevention of chronic pain after surgical nerve injury: amputation and thoracotomy. Surg Clin North Am 92: 393–x, 393-407, x. 22414418 10.1016/j.suc.2012.01.005

[3] Enck RE (2010) Postsurgical chronic pain. Am J Hosp Palliat Care 27: 301–302. 20466940 10.1177/1049909110369530

[4] Haroutiunian S, Nikolajsen L, Finnerup NB, Jensen TS (2013) The neuropathic component in persistent postsurgical pain: a systematic literature review. Pain 154: 95–102. 23273105 10.1016/j.pain.2012.09.010

[5] Van de Ven TJ, John Hsia HL (2012) Causes and prevention of chronic postsurgical pain. Curr Opin Crit Care 18: 366–371. 22732437 10.1097/MCC.0b013e3283557a7f

[6] Guastella V, Mick G, Soriano C, Vallet L, Escande G, et al (2011) A prospective study of neuropathic pain induced by thoracotomy: incidence, clinical description, and diagnosis. Pain 152: 74–81. 21075523 10.1016/j.pain.2010.09.004

[7] Katz J, Jackson M, Kavanagh BP, Sandler AN (1996) Acute pain after thoracic surgery predicts long-term post-thoracotomy pain. Clin J Pain 12: 50–55. 8722735 10.1097/00002508-199603000-00009

[8] Steegers MA, Snik DM, Verhagen AF, van der Drift MA, Wilder-Smith OH (2008) Only half of the chronic pain after thoracic surgery shows a neuropathic component. J Pain 9: 955–961. 18632308 10.1016/j.jpain.2008.05.009

[9] Deumens R, Steyaert A, Forget P, Schubert M, Lavand'homme P, et al (2013) Prevention of chronic postoperative pain: cellular, molecular, and clinical insights for mechanism-based treatment approaches. Prog Neurobiol 104: 1–37. 23410739 10.1016/j.pneurobio.2013.01.002

[10] International Association for the Study of Pain Committee. ASP Taxonomy. Pain terms — Changes in the 2011 list. Available from www.iasp-pain.org/Content/NavigationMenu/GeneralResourceLinks/PainDefinitions/default.htm. Accessed 2012 Feb 18

[11] Searle RD, Simpson MP, Simpson KH, Milton R, Bennett MI (2009) Can chronic neuropathic pain following thoracic surgery be predicted during the postoperative period? Interact Cardiovasc Thorac Surg 9: 999–1002. 19767301 10.1510/icvts.2009.216887

[12] Maguire MF, Ravenscroft A, Beggs D, Duffy JP (2006) A questionnaire study investigating the prevalence of the neuropathic component of chronic pain after thoracic surgery. Eur J Cardiothorac Surg 29: 800–805. 16581259 10.1016/j.ejcts.2006.02.002

[13] Salati M, Brunelli A, Xiume F, Refai M, Sabbatini A (2009) Quality of life in the elderly after major lung resection for lung cancer. Interact Cardiovasc Thorac Surg 8: 79–83. 18940832 10.1510/icvts.2008.184986

[14] Brunelli A, Socci L, Refai M, Salati M, Xiume F, et al (2007) Quality of life before and after major lung resection for lung cancer: a prospective follow-up analysis. Ann Thorac Surg 84: 410–416. 17643607 10.1016/j.athoracsur.2007.04.019

[15] Jensen TS, Baron R, Haanpaa M, Kalso E, Loeser JD, et al (2011) A new definition of neuropathic pain. Pain 152: 2204–2205. 21764514 10.1016/j.pain.2011.06.017

[16] Haanpaa M, Attal N, Backonja M, Baron R, Bennett M, et al (2011) NeuPSIG guidelines on neuropathic pain assessment. Pain 152: 14–27. 20851519 10.1016/j.pain.2010.07.031

[17] Li J, Feng Y, Han J, Fan B, Wu D, et al (2012) Linguistic adaptation, validation and comparison of 3 routinely used neuropathic pain questionnaires. Pain Physician 15: 179–186. 22430656

[18] Wildgaard K, Ravn J, Kehlet H (2009) Chronic post-thoracotomy pain: a critical review of pathogenic mechanisms and strategies for prevention. Eur J Cardiothorac Surg 36: 170–180. 19307137 10.1016/j.ejcts.2009.02.005

[19] Perttunen K, Tasmuth T, Kalso E (1999) Chronic pain after thoracic surgery: a follow-up study. Acta Anaesthesiol Scand 43: 563–567. 10342006 10.1034/j.1399-6576.1999.430513.x

[20] VanDenKerkhof EG, Peters ML, Bruce J (2013) Chronic pain after surgery: time for standardization? A framework to establish core risk factor and outcome domains for epidemiological studies. Clin J Pain 29: 2–8. 23211602 10.1097/AJP.0b013e31824730c2

[21] Macrae WA (2001) Chronic pain after surgery. Br J Anaesth 87: 88–98. 11460816 10.1093/bja/87.1.88

[22] Wang HT, Liu W, Luo AL, Ma C, Huang YG (2012) Prevalence and risk factors of chronic post-thoracotomy pain in Chinese patients from Peking Union Medical College Hospital. Chin Med J (Engl) 125: 3033–3038. 22932175

[23] Mongardon N, Pinton-Gonnet C, Szekely B, Michel-Cherqui M, Dreyfus JF, et al (2011) Assessment of chronic pain after thoracotomy: a 1-year prevalence study. Clin J Pain 27: 677–681. 21705876 10.1097/AJP.0b013e31821981a3

[24] Song JG, Shin JW, Lee EH, Choi DK, Bang JY, et al (2012) Incidence of post-thoracotomy pain: a comparison between total intravenous anaesthesia and inhalation anaesthesia. Eur J Cardiothorac Surg 41: 1078–1082. 22290901 10.1093/ejcts/ezr133

[25] Lavand'homme P (2011) The progression from acute to chronic pain. Curr Opin Anaesthesiol 24: 545–550. 21772141 10.1097/ACO.0b013e32834a4f74

[26] Portenoy R (2006) Development and testing of a neuropathic pain screening questionnaire: ID Pain. Curr Med Res Opin 22: 1555–1565. 16870080 10.1185/030079906X115702

[27] Reyes-Gibby C, Morrow PK, Bennett MI, Jensen MP, Shete S (2010) Neuropathic pain in breast cancer survivors: using the ID pain as a screening tool. J Pain Symptom Manage 39: 882–889. 20471548 10.1016/j.jpainsymman.2009.09.020 PMC2872632

[28] Wu CL, Raja SN (2011) Treatment of acute postoperative pain. Lancet 377: 2215–2225. 21704871 10.1016/S0140-6736(11)60245-6

[29] Ochroch EA, Gottschalk A, Troxel AB, Farrar JT (2006) Women suffer more short and long-term pain than men after major thoracotomy. Clin J Pain 22: 491–498. 16772805 10.1097/01.ajp.0000208246.18251.f2

[30] Puolakka PA, Rorarius MG, Roviola M, Puolakka TJ, Nordhausen K, et al (2010) Persistent pain following knee arthroplasty. Eur J Anaesthesiol 27: 455–460. 20299989 10.1097/EJA.0b013e328335b31c

[31] Wildgaard K, Ravn J, Nikolajsen L, Jakobsen E, Jensen TS, et al (2011) Consequences of persistent pain after lung cancer surgery: a nationwide questionnaire study. Acta Anaesthesiol Scand 55: 60–68. 21077845 10.1111/j.1399-6576.2010.02357.x

[32] Racine M, Tousignant-Laflamme Y, Kloda LA, Dion D, Dupuis G, et al (2012) A systematic literature review of 10 years of research on sex/gender and pain perception - part 2: do biopsychosocial factors alter pain sensitivity differently in women and men? Pain 153: 619–635. 22236999 10.1016/j.pain.2011.11.026

[33] Mogil JS, Bailey AL (2010) Sex and gender differences in pain and analgesia. Prog Brain Res 186: 141–157. 21094890 10.1016/B978-0-444-53630-3.00009-9

[34] Kehlet H, Jensen TS, Woolf CJ (2006) Persistent postsurgical pain: risk factors and prevention. Lancet 367: 1618–1625. 16698416 10.1016/S0140-6736(06)68700-X

[35] Pluijms WA, Steegers MA, Verhagen AF, Scheffer GJ, Wilder-Smith OH (2006) Chronic post-thoracotomy pain: a retrospective study. Acta Anaesthesiol Scand 50: 804–808. 16879462 10.1111/j.1399-6576.2006.01065.x

[36] Gotoda Y, Kambara N, Sakai T, Kishi Y, Kodama K, et al (2001) The morbidity, time course and predictive factors for persistent post-thoracotomy pain. Eur J Pain 5: 89–96. 11394926 10.1053/eujp.2001.0225

[37] Katz J, Seltzer Z (2009) Transition from acute to chronic postsurgical pain: risk factors and protective factors. Expert Rev Neurother 9: 723–744. 19402781 10.1586/ern.09.20

[38] Kalso E, Perttunen K, Kaasinen S (1992) Pain after thoracic surgery. Acta Anaesthesiol Scand 36: 96–100. 1539485 10.1111/j.1399-6576.1992.tb03430.x

[39] Tiippana E, Nilsson E, Kalso E (2003) Post-thoracotomy pain after thoracic epidural analgesia: a prospective follow-up study. Acta Anaesthesiol Scand 47: 433–438. 12694143 10.1034/j.1399-6576.2003.00056.x

[40] Khan IH, McManus KG, McCraith A, McGuigan JA (2000) Muscle sparing thoracotomy: a biomechanical analysis confirms preservation of muscle strength but no improvement in wound discomfort. Eur J Cardiothorac Surg 18: 656–661. 11113671 10.1016/s1010-7940(00)00591-1

[41] American Society of Anesthesiologists Task Force on Acute Pain M (2012) Practice guidelines for acute pain management in the perioperative setting: an updated report by the American Society of Anesthesiologists Task Force on Acute Pain Management. Anesthesiology 116: 248–273. 22227789 10.1097/ALN.0b013e31823c1030

[42] Wildgaard K, Kehlet H (2011) Chronic post-thoracotomy pain—What is new in pathogenic mechanisms and strategies for prevention? Techniques in Regional Anesthesia and Pain Management 15: 83–89.

[43] Wildgaard K, Ringsted TK, Ravn J, Werner MU, Kehlet H (2013) Late sensory changes following chest drain insertion during thoracotomy. Acta Anaesthesiol Scand 57: 776–783. 23379676 10.1111/aas.12077

[44] Miyazaki T, Sakai T, Yamasaki N, Tsuchiya T, Matsumoto K, et al (2013) Chest tube insertion is one important factor leading to intercostal nerve impairment in thoracic surgery. Gen Thorac Cardiovasc Surg 10.1007/s11748-013-0328-z 24096982

[45] Forcella D, Pompeo E, Coniglione F, Gatti A, Mineo TC (2009) A new technique for continuous intercostal-intrapleural analgesia in videothoracoscopic surgery. J Thorac Cardiovasc Surg 137: e48–49. 19154885 10.1016/j.jtcvs.2008.03.062

[46] Nakamura H, Taniguchi Y, Miwa K, Adachi Y, Fujioka S, et al (2009) The use of Blake drains following general thoracic surgery: is it an acceptable option? Interact Cardiovasc Thorac Surg 8: 58–61. 18835856 10.1510/icvts.2008.188086

